# Release of Terpenes from Fir Wood during Its Long-Term Use and in Thermal Treatment

**DOI:** 10.3390/molecules17089990

**Published:** 2012-08-21

**Authors:** František Kačík, Veronika Veľková, Pavel Šmíra, Andrea Nasswettrová, Danica Kačíková, Ladislav Reinprecht

**Affiliations:** 1 Department of Chemistry and Chemical Technologies, Faculty of Wood Sciences and Technology, Technical University in Zvolen, T. G. Masaryka 24, 960 53 Zvolen, Slovakia; 2 Thermo Sanace, Chamrádová 475/23, 718 00 Ostrava-Kunčičky, Czech Republic; 3 Department of Fire Protection, Faculty of Wood Sciences and Technology, Technical University in Zvolen, T. G. Masaryka 24, 960 53 Zvolen, Slovakia; 4 Department of Mechanical Wood Technology, Faculty of Wood Sciences and Technology, Technical University in Zvolen, T. G. Masaryka 24, 960 53 Zvolen, Slovakia

**Keywords:** terpenes, fir wood, thermal treatment, old wood

## Abstract

Building structures made from fir wood are often attacked by wood-destroying insects for which the terpenes it contains serve as attractants. One of the possibilities for extending the lifetime of structures is to use older wood with a lower content of terpenes and/or thermally modified wood. The study evaluated the levels of terpenes in naturally aged fir wood (108, 146, 279, 287 and 390 years) and their decrease by thermal treatment (the temperature of 60 °C and 120 °C, treatment duration of 10 h). Terpenes were extracted from wood samples by hexane and analyzed by gas-chromatography mass-spectrometry (GC-MS). The results indicate that recent fir wood contained approximately 60 times more terpenes than the oldest wood (186:3.1 mg/kg). The thermal wood treatment speeded up the release of terpenes. The temperature of 60 °C caused a loss in terpenes in the recent fir wood by 62%, the temperature of 120 °C even by >99%. After the treatment at the temperature of 60 °C the recent fir wood had approximately the same quantity of terpenes as non-thermally treated 108 year old wood, *i.e.*, approximately 60–70 mg/kg. After the thermal treatment at the temperature of 120 °C the quantity of terpenes dropped in the recent as well as the old fir wood to minimum quantities (0.7–1.1 mg/kg). The thermal treatment can thus be used as a suitable method for the protection of fir wood from wood-destroying insects.

## 1. Introduction

There are several types of monoterpenes in fir wood, particularly α-pinene, β-pinene, limonene, Δ3-carene, camphene, β-phellandrene and myrcene. Monoterpenes are acyclic, monocyclic and/or bicyclic C-10 isoprenoides structurally derived from isoprene. They are natural products with characteristic odours that affect the growth regulation, reproduction cycles, defence mechanisms and the transmission of signals of various organisms. Some monoterpenes have toxic effects on fungi and insects, and they seem to be essential components of conifer resistance due to their physically repellent and chemically toxic properties [1,2]. Conifer terpenoid profiles vary geographically [3], across closely related taxa [4] and among genotypes of the same species [5]. Within a species, several examples exist of differences in tree resistance to specific insects, and some of these correlate with terpenoid profiles [2]. For example, limonene and other terpenes correlate with resistance of Italian stone pine (*Pinus pinea:* Pinaceae) and Aleppo pine (*Pinus halepensis*) to attack by the scale insect *Marchalina hellenica* (Homoptera) [6]. Bornyl acetate [7] and other terpenes [8] are important in the interaction between the western spruce budworm (*Coristoneura occidentalis:* Lepidoptera) and its Douglas-fir host. However, other monoterpenes can act as attractants for some insect species [9,10,11,12]. Fettköther *et al.* [13] found out that females of the wood-destroying beetle—house longhorn beetle (*Hylotrupes bajulus*) are attracted by a mixture of (−)-verbenone, (+)-α-pinene, (+)-terpinen-4-ol and (−)-trans-pinocarveol, while males are attracted by a mixture of (+)-terpinen-4-ol, (+)-α-pinene and (−)-trans-pinocarveol. 

Wood is currently being used more and more for construction purposes due to its advantageous properties (strength, flexibility, thermal and sound insulation). On the other hand, it also shows some disadvantages such as flammability and low resistance to fungi and insects. It is said in the construction practice that log houses, roof timber, ceilings and others structures made of soft wood are more resistant against an attack by wood-destroying insects if they are produced from older seasoned wood from which monoterpenes (or higher terpenes as well) have vaporised and/or transformed into other compounds. In contrast, structures made of fresh timber with higher levels of monoterpenes are more liable to the attacks of *H. bajulus* beetles as well as other species of wood-destroying insects. Terpenes are released from wood during its storage and its first-stage processing—debarking, sawing, peeling, cutting, chipping and drying [14,15,16]. 

For the purpose of extending the lifetime of wooden parts of newly built houses and historical buildings, various methods for their protection have been studied such as wood-destroying beetle traps [17,18], chemical wood treatment with insecticides [19,20] and/or thermal modification of wood at the temperature of 160–220 °C which improves its dimensional stability and biological resistance [21,22]. A more moderate form of thermal wood protection is its thermal sterilisation—so called “thermal remediation”. In thermal remediation, wood is treated with air heated to the temperature of 120 °C, where it is necessary to achieve at least the temperature of 55–60 °C in the wood cross-section for the period of 1 h. It is sufficient for the coagulation of proteins and killing wood-destroying insects and at the same time it reduces the quantity of terpenes present in wood which is then less attractive to insects. The thermal sterilisation utilisation is mainly suitable for historical buildings, where the application of fungicides and insecticides is impossible.

To isolate terpenes from various matrices (needles, leaves, wood, honey, wine *etc.*), various technologies are used such as water steam distillation, supercritical carbon dioxide distillation, solid-phase extraction (SPE), solid-phase micro-extraction (SPME) and extraction using solvents—hexane, dichloromethane, acetone, ether, methanol. In this study, the isolation method of the extraction with hexane was used. Gas-chromatography (GC) and/or the combination of gas-chromatography and mass-spectroscopy (GC-MS) are commonly used for terpene determination [23,24,25,26,27,28,29]. The method of GC-MS was used in this study. 

The quantity and composition of terpenes in wood depends on the wood species, growth location, harvest season, *etc.* In fir wood in particular α-pinene, β-pinene, limonene, Δ^3^-carene, camphene, β-phellandrene, myrcene are found, in various concentration ranging from trace quantities to dozens of percents depending on the species and location [26,30,31,32]. The wood of coniferous trees contains approximately 0.1%–0.6% of terpenes. In needles, approximately five times higher concentrations were found compared to wood [3,33,34]. The rate of release of terpenes from harvested wood depends on the size of samples, moisture content, temperature, the manner of further processing and the course of exposure. A higher temperature significantly speeds up emissions of terpenes from wood. This is shown at different manners of drying, when there are several mechanisms of movement and release of terpenes from wood [35]. McDonald and Wastney [36] found out about 60%–70% higher emissions of volatile organic compound (VOC) at the temperature of 140 °C than at 120 °C. In contrast, Ingram *et al.* [37] did not observe any differences in VOC emissions between the temperatures of 82 °C and 118 °C. Banerjee [35] found an increase of α-pinene emissions with an increasing temperature in the temperature range of 105–200 °C. Peters *et al.* [16] determined about a 30% decrease in the amount of terpenes in spruce wood during its treatment at the temperature of 180 °C, but they did not observe any further drop of their concentration at 200 °C. The thermal treatment conditions used have no adverse effect on mechanical wood properties [38] and therefore they can be applied as a suitable ecological and economic alternative for its sterilisation and long-term protection.

Data on changes in terpenes in wood after its long-lasting use as a building material and on the impact of thermal sterilisation on such wood are missing in scientific literature. The aim of this paper was to evaluate changes in the quantities of selected terpenes in fir wood depending on the age of building structures as well as on conditions of thermal sterilisation—wood treatment. The obtained results can be useful to the thermal sterilisation of buildings attacking by wood-borer insect and/or at the thermal treatment of wood dedicated to building purposes.

## 2. Results and Discussion

In the samples of fir wood we found α-pinene, camphene, β-pinene, α-phellandren, cymene, limonene, fenchol, borneol, thymol, myrtenal and verbenone (Table 1).

**Table 1:** Quantities of terpenes in samples of non-treated wood (mg/kg).

Components	A (1622)	B (1725)	C (1733)	D (1866)	E (1904)	F (2011)
**α-Pinene ^1^**	0.5591 ± 0.011	0.9940 ± 0.020	5.5471 ± 0.017	4.3292 ± 0.026	34.8298 ± 0.008	115.4721 ± 1.095
**Camphene ^1^**	-	-	-	0.3871 ± 0.004	5.0133 ± 0.042	12.3156 ± 0.297
**β-Pinene ^1^**	0.2759 ± 0.015	0.2989 ± 0.004	0.5565 ± 0.012	0.1918 ± 0.003	0.5448 ± 0.025	20.9857 ± 0.087
**α-Phellandrene ^1^**	0.2989 ± 0.023	0.3795 ± 0.011	0.2715 ± 0.014	-	1.4577 ± 0.029	1.6450 ± 0.041
**Cymene ^2^**	0.0000 ± 0.000	0.6989 ± 0.013	0.0000 ± 0.000	-	2.0209 ± 0.03	6.1589 ± 0.047
**Limonene ^1^**	1.0155 ± 0.026	2.0284 ± 0.015	1.1708 ± 0.008	1.0672 ± 0.013	1.5015 ± 0.021	6.1684 ± 0.120
**Fenchol ^2^**	-	-	-	-	0.5582 ± 0.007	2.3811 ± 0.172
**Borneol ^2^**	-	-	-	-	1.0892 ± 0.017	3.3375 ± 0.079
**Thymol ^2^**	-	0.2284 ± 0.002	-	0.5200 ± 0.014	1.2312 ± 0.011	4.3070 ± 0.127
**Myrtenal ^2^**	0.5330 ± 0.013	0.8778 ± 0,005	0.5144 ± 0.013	1.2875 ± 0.004	5.1788 ± 0.02	6.1012 ± 0.214
**Verbenon ^2^**	0.4081 ± 0.010	0.4279 ± 0.014	-	0.7923 ± 0.019	5.2141 ± 0.138	7.6208 ± 0.134
**Total**	**3.0906** ± 0.071	**5.9338** ± 0.045	**8.0603** ± 0.027	**8.5751** ± 0.058	**58.6394** ± 0.214	**186.4933** ± 1.943

Note: ^1^ identified by MS-database and retention time data; ^2^ identification was based on MS-database only.

Quantities of individual and total terpenes determined by us in fir beams are lower compared to their amounts in fir wood of freshly cut trees [26,31]. The reason is a partial evaporation of terpenes during the processing of fresh logs into timber (sawing, drying *etc.*) and as a result of their long-term use as stated in Granström [39]. It follows from the data in Table 1 that the amount of terpenes in fir wood decreases with the age of the used material. The decrease of the total terpene amount achieved the highest value between the current wood sample (F = 186 mg/kg) and a 150 years old wood sample (D = 8.6 mg/kg). Most terpenes were thus released during the first years of fir beam exposure in buildings, while later the rate of release decreased and their total amount in beams approached the limit value of 3 mg/kg (Sample A). Resulting values are similar to those found by Granström [33,34] and Rupar, Sanati [15] during the storage of fir wood chips at various levels of moisture content. 

**Figure 1:**
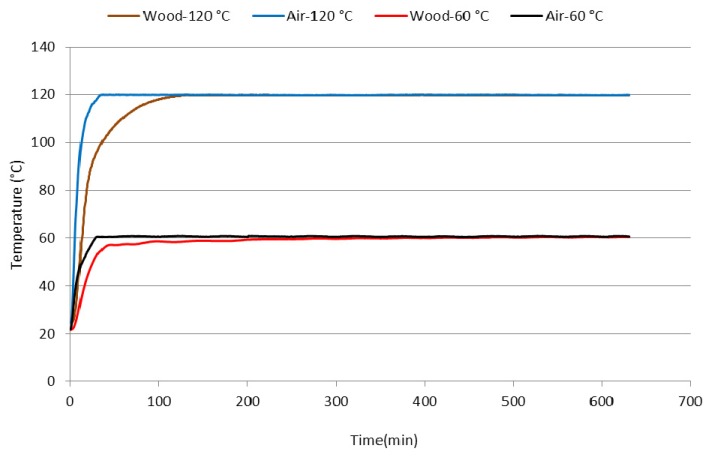
Temperatures in the dryer and in samples during the thermal treatment at 60 °C and 120 °C.

For thermal sterilisation treatment of wood (thermal remediation), hot air at a temperature of 120 °C is used which affects the surface of wooden elements with the goal of achieving a temperature between 55 and 60 °C inside. Due to the quite big dimensions of wooden elements in roofs and other structures as well as heat leakage from reconstructed buildings the heat transmission to bigger elements is quite slow and the whole process of thermal remediation lasts for 10–24 h. For this reason, two temperatures operating for 10 h were used under laboratory conditions in the thermal treatment of fir wood samples: (a) 60 °C—the temperature inside wood if the heating of small cross-section elements such as battens is modelled; (b) 120 °C—the temperature on wood surface during a practical thermal remediation of buildings (Figure 1).

An increased temperature causes faster release of terpenes into air which results in their lower concentration in the thermally treated sample (Table 2 and Table 3). The temperature of 60 °C caused a decrease in the quantity of terpenes in the recent wood (Sample F) by 62%, and the temperature of 120 °C by >99%. Results of other authors differ depending on the temperature and the form of its action. In agreement with McDonald and Wastney [36] we observed increased emissions of terpenes at the higher temperatures.

**Table 2:** Quantities of terpenes in samples of treated wood at the temperature of 60 °C (mg/kg).

Components	A (1622)	B (1725)	C (1733)	D (1866)	E (1904)	F (2011)
**α-Pinene ^1^**	0.2766 ± 0.003	-	0.5243 ± 0.023	2.4906 ± 0.018	24.7044 ± 0.139	43.5522 ± 0.035
**Camphene ^1^**	-	-	0.7200 ± 0.024	-	4.1916 ± 0.098	8.4603 ± 0.026
**ß-Pinene ^1^**	0.0506 ± 0.003	-	-	0.3041 ± 0.011	0.4841 ± 0.019	0.8930 ± 0.012
**α-Phellandrene ^1^**	-	-	-	0.3971 ± 0.013	1.4752 ± 0.017	0.9597 ± 0.008
**Cymene ^2^**	-	-	-	0.5163 ± 0.015	1.3008 ± 0.058	3.8669 ± 0.028
**Limonene ^1^**	0.8267 ± 0.012	0.6107 ± 0.011	0.6847 ± 0.011	2.3443 ± 0.004	0.8725 ± 0.05	0.7227 ± 0.003
**Fenchol ^2^**	-	-	-	-	0.6654 ± 0.014	0.5609 ± 0.007
**Borneol ^2^**	-	-	-	-	1.2818 ± 0.020	1.2658 ± 0.024
**Thymol ^2^**	0.2809 ± 0.015	0.2492 ± 0.032	0.3240 ± 0.028	0.2441 ± 0.016	1.3113 ± 0.080	3.3447 ± 0.009
**Myrtenal ^2^**	0.6276 ± 0.014	0.5774 ± 0.005	0.8914 ± 0.055	1.0085 ± 0.017	4.8605 ± 0.11	3.0267 ± 0.056
**Verbenon ^2^**	-	0.2620 ± 0.030	0.2965 ± 0.020	0.3579 ± 0.020	8.4572 ± 0.166	3.1520 ± 0.086
**Total**	**2.0625** ± 0.004	**1.6993** ± 0.069	**3.4410** ± 0.101	**7.6630** ± 0.081	**49.6046 **± 0.239	**69.8048** ± 0.076

Note: ^1^ identified by MS-database and retention time data; ^2^ identification was based on MS-database only.

**Table 3:** Quantities of terpenes in samples of treated wood at the temperature of 120 °C (mg/kg).

Components	A (1622)	B (1725)	C (1733)	D (1866)	E (1904)	F (2011)
**α-Pinene ^1^**	0.1141 ± 0.001	-	-	-	0.0826 ± 0.0006	0.1687 ± 0.0034
**Camphene ^1^**	-	-	-	-	0.0547 ± 0.0007	0.1746 ± 0.0033
**ß-Pinene ^1^**	0.0291 ± 0.002	-	-	-	0.0033 ± 0.0002	0.0059 ± 0.0002
**α-Phellandrene ^1^**	-	-	-	-	0.0102 ± 0.0002	0.0077 ± 0.0001
**Cymene ^2^**	-	-	-	-	0.0198 ± 0.0009	0.0672 ± 0.0013
**Limonene ^1^**	0.1089 ± 0.001	0.4629 ± 0.005	0.5499 ± 0.014	0.6303 ± 0.006	0.0103 ± 0.0004	0.0163 ± 0.0002
**Fenchol ^2^**	0.0440 ± 0.004	-	-	-	0.0076 ± 0.0002	0.0399 ± 0.0022
**Borneol ^2^**	-	-	-	-	0.0116 ± 0.0004	0.0459 ± 0.0009
**Thymol ^2^**	0.2737 ± 0.011	-	-	-	0.0089 ± 0.0001	0.0252 ± 0.0009
**Myrtenal ^2^**	0.1271 ± 0.003	0.2892 ± 0.006	0.3147 ± 0.007	0.3373 ± 0.006	0.0353 ± 0.0008	0.0413 ± 0.0004
**Verbenon ^2^**	-	-	-	-	0.2145 ± 0.0037	0.4551 ± 0.0086
**Total**	**0.6969 **± 0.021	**0.7521** ± 0.002	**0.8646** ± 0.019	**0.9676** ± 0.001	**0.4588** ± 0.007	**1.0480** ± 0.0199

Note: ^1^ identified by MS-database and retention time data; ^2^ identification was based on MS-database only.

**Figure 2:**
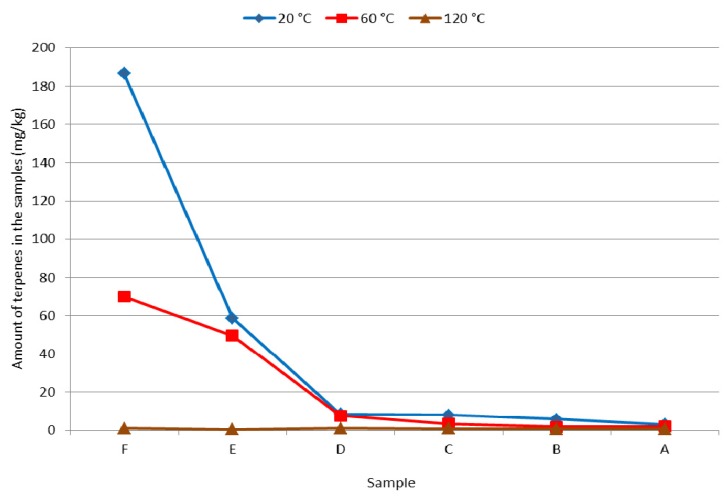
Total quantity of terpenes in original and thermally treated fir wood samples of different ages (mg/kg).

The decrease of terpenes in fir wood samples exposed in structures for a long time was milder after its thermal treatment (Table 1, Table 2, Table 3, Figure 2). In addition to the total quantity of terpenes, wood resistance against wood-destroying insects has an impact on concentrations of β-pinene and the ratio of β-pinene/α-pinene [40], as α-pinene increases the activity and orientation of the beetle *H. bajulus* [13]. The pheromones verbenone and *p*-cymen-8-ol produced by insect larvae of *H. bajulus* also supported egg laying [41]. The wood thermal sterilisation during which the temperature of at least 60 °C is achieved in the whole cross-section of used parts guarantees killing all live forms of wood-destroying insects (eggs, larvae, adult individuals), and at the same time the quantity of terpenes in wood which can serve as attractants for these insects is reduced. 

## 3. Experimental

### 3.1. Material

Drill hole samples were taken from fir wood (*Abies alba* Mill.) beams situated in five historical buildings and one newly built building and on this basis, the age of beams was determined using a dendrochronological method (years of origin: A—1622, B—1725, C—1733, D—1866, E—1904, F—2011). Subsequently, samples with dimensions of 20 × 20 × 40 mm were taken from the beams. These either were and/or were not thermally treated in a Memmert UNB 200 drier (Memmert GmbH, Schwabach, Germany) at the temperatures of 60 °C, resp. 120 °C for 10 h. Temperatures in the drier and in the centre of individual samples were measured by NiCr-Ni thermocouples with the thickness of 0.4 mm using an Almemo 2290-8 device (Ahlborn, Holzkirchen, Germany). The samples of thermally non-treated and treated samples were disintegrated to sawdust in a Polymix PX-MFC 90D mill (Kinematica AG, Luzern, Switzerland) and sieved to individual fractions (<0.35 mm, 0.35–0.50 mm, 0.50–1.00 mm, >1.00 mm) in Analysette 3 (Fritsch, Idar-Oberstein, Germany).

### 3.2. Extraction

Sawdust fractions of 0.35–0.50 mm (10 g) were extracted by hexane (100 mL) in a Promax 2020 shaker (Heidolph, Kelheim, Germany) for 24 h, party evaporated under vacuum at the temperature of 27 °C and the final sample amount (1 mL) was obtained in a gentle nitrogen flow. Two independent extractions were performed, and each extract was injected twice into the GC-MS.

### 3.3. Chromatographic Analysis

The GC-MS analyses of extracted terpenes were performed using an HP 7890A-5975C VL MSD instrument (Agilent Technologies, Santa Clara, CA, USA) equipped with an HP-5 MS column (30 m × 250 µm i.d., 0.25 µm film thickness) (Agilent J&W). Helium was used as the carrier gas at 1.5 mL·min^−1^ flow rate. The temperature program was starting at the temperature 75 °C (5 min hold), and then increased to 140 °C at 7 °C·min^−1^, followed by a 20 °C·min^−1^ ramp until 250 °C (5 min hold). The split-splitless injector and MS-transfer line were held at 250 °C and 280 °C, respectively. MSD was operated in electron impact ionization mode at 70 eV electron energy. The injected volume was 1 µL and the split ration was 1:20. Compounds were identified by comparing mass spectra using Agilent Technologies software and NIST05 MS-library, as well as by comparing retention times and mass spectra with those of authentic standards. Concentrations were quantified by means of peak areas by using standard compounds for calibration.

## 4. Conclusions

Terpenes in fir wood after its long-term use as a building material are released especially during first 150 years of use. Fir wood thermal modification at the temperature 60 °C accelerates the terpene emission and at the temperature 120 °C removes the terpenes almost completely. The thermal treatment can thus be used as a suitable method for the protection of fir wood from wood-destroying insects, as it decreases the amount of terpenes which are insect attractants for more insect-species and parallely kills the actual insects in attacked wood. In our next research the studies on biological activity against insects (attractant/repellent) will be carried out.

## References

[1] Franceschi V.R., Krokene P., Christiansen E., Krekling T. (2005). Anatomical and chemical defences of conifer bark beetles and other pests. New Phytol..

[2] Keeling C.I., Bohlmann J. (2006). Genes, enzymes and chemicals of terpenoid diversity in the constitutive and induced defence of conifers against insects and pathogens. New Phytol..

[3] Manninen A.M., Tarhanen S., Vuorinen M., Kainulainen P. (2002). Comparing the variation of needle and wood terpenoids in scots pine provenances. J. Chem. Ecol..

[4] Fäldt J., Sjodin K., Persson M., Valterova I., Borg-Karlson A.K. (2001). Correlations between selected monoterpene hydrocarbons in the xylem of six *Pinus* (Pinaceae) species. Chemoecology.

[5] Martin D.M., Gershenzon J., Bohlmann J. (2003). Induction of volatile terpene biosynthesis and diurnal emission by methyl jasmonate in foliage of Norway spruce. Plant Physiol..

[6] Mita E., Tsitsimpikou C., Tsiveleka L., Petrakis P.V., Ortiz A., Vagias C., Roussis V. (2002). Seasonal variation of oleoresin terpenoids from *Pinus halepensis* and *Pinus pinea* and host selection of the scale insect *Marchalina hellenica* (Homoptera, Coccoidea, Margarodidae, Coelostonidiinae). Holzforschung.

[7] Cates R.G., Henderson C.B., Redak R.A. (1987). Responses of the western spruce budworm to varying levels of nitrogen and terpenes. Oecologia.

[8] Chen Z., Kolb T.E., Clancy K.M. (2002). The role of monoterpenes in resistance of Douglas fir to western spruce budworm defoliation. J. Chem. Ecol..

[9] Harborne J.B. (1988). Introduction to Ecological Biochemistry.

[10] Holubová V., Hrdlička P., Kubáň V. (2001). Age and space distributions of monoterpenes in fresh needles of *Picea abies* (L) Karst. determined by gas chromatography-mass spectrometry. Phytochem. Anal..

[11] Singer A.C., Crowley D.E., Thompson I.P. (2003). Secondary plant metabolites in phytoremediation and biotransformation. Trends Biotechnol..

[12] Dvořáková M., Valterová I., Vaněk T. (2011). Monoterpenes in plants. Chem. Listy.

[13] Fettköther R., Reddy G.V.P., Noldt U., Dettner K. (2000). Effect of host and larval frass volatiles on behavioural response of the old-house borer *Hylotrupes bajulus* (L.) (Coleoptera: Cerambycidae) in a wind tunnel bioassay. Chemoecology.

[14] Granström K.M. (2003). Emissions of monoterpenes and VOC during drying of sawdust in a continuous spouted bed. Forest Prod. J..

[15] Rupar K., Sanati M. (2005). The release of terpenes during storage of biomass. Biomass Bioenerg..

[16] Peters J., Fischer K., Fischer S. (2008). Characterization of emissions from thermally modified wood and their reduction by chemical treatment. Bioresources.

[17] Reddy G.V.P., Fettköther R., Noldt U., Dettner K. (2005). Enhancement of attraction and trap catches of the old-house borer, *Hylotrupes bajulus* (Coleoptera: Cerambycidae), by combination of male sex pheromone and monoterpenes. Pest Manag. Sci..

[18] Reddy G.V.P. (2007). Improved semiochemical-based trapping method for old-house borer, *Hylotrupes bajulus* (Coleoptera: Cerambycidae). Environ. Entomol..

[19] Hertel H. (1997). Protection of wood against the house longhorn beetle *Hylotrupes bajulus* with sodium chloride and potassium chloride. Pestic. Sci..

[20] Hagle J.R., Blackmer J.L. (2007). Potassium chloride deters *Lygus hesperus* feeding behavior. Entomol. Exp. Appl..

[21] Esteves B., Domingos I., Pereira H. (2008). Pine wood modification by heat treatment in air. Bioresources.

[22] Esteves B., Pereira H. (2009). Wood modification by heat treatment: A review. Bioresources.

[23] Muzika R.-M., Campbell C.L., Hanover J.W., Smith A.L. (1990). Comparison of techniques for extracting volatile compounds from conifer needles. J. Chem. Ecol..

[24] Bowman J.M., Braxton M.S., Churchill M.A., Hellie J.D., Starrett S.J., Causby G.Y., Ellis D.J., Ensley S.D., Maness S.J., Meyer C.D. (1997). Extraction method for the isolation of terpenes from plant tissue and subsequent determination by gas chromatography. Microchem. J..

[25] Martin-Benito D., Garcia-Vallejo M.C., Pajares J.A., López D. (2005). Triterpenes in elms in Spain. Can. J. For. Res..

[26] Tumen I., Hafizoglu H., Kilic A., Dönmez I.E., Sivrikaya H., Reunanen M. (2010). Yields and constituents of essential oil from cones of Pinaceae spp. natively grown in Turkey. Molecules.

[27] Jerković I., Marijanović Z., Malenica-Staver M., Lušić D. (2010). Volatiles from a rare acer spp. honey sample from Croatia. Molecules.

[28] Gao B., Chen Y., Zhang M., Xu Y., Pan S. (2011). Chemical composition, antioxidant and antimicrobial activity of *Pericarpium Citri Reticulatae* essential oil. Molecules.

[29] Tadić V., Bojović D., Arsić I., Dordević S., Aksentijevic K., Stamenić M., Janković S. (2012). Chemical and antimicrobial evaluation of supercritical and conventional *Sideritis scardica* griseb., lamiaceae extracts. Molecules.

[30] Čermák J. (1987). Monoterpene hydrocarbon contents of the resin from seeds of silver fir (*Abies alba* Mill.). Trees Struct. Funct..

[31] Sutton B.A., Woosley R.S., David J., Butcher D.J. (1997). Determination of monoterpenes in oleoresin: A chemosystematic study of the interaction between fraser fir (*Abies fraseri*) and balsam woolly adelgid (*Adelges piceae*). Microchem. J..

[32] Fengel D., Wegener G. (2003). Wood: Chemistry, Ultrastructure, Reactions.

[33] Granström K.M. (2005). Emissions of Volatile Organic Compounds from Wood. Dissertation.

[34] Granström K.M., Borrego C.A., Brebbia C.A. (2007). Wood Processing as a Source of Terpene Emissions Compared to Natural Sources. Air Polution XV.

[35] Banerjee S. (2001). Mechanism of terpene release during sawdust and flake drying. Holzforschung.

[36] McDonald A.G., Wastney S. Analysis of Volatile Emissions from Kiln Drying of Radiata Pine. Proceedings of the 8th International Symposium on Wood and Pulping Chemistry.

[37] Ingram L., Taylor F., Templeton M. (1996). Volatile Organic Compound Emissions from Southern Pine Kilns. Proceedings of the Drying Pacific Northwest Species for Quality Markets.

[38] Reinprecht L., Vidholdová Z. (2011). Termodrevo—Thermowood.

[39] Granström K.M. (2009). Emissions of sesquiterpenes from spruce sawdust during drying. Eur. J. Wood Prod..

[40] Nerg A.M., Heijari J., Noldt U., Viitanen H., Vuorinen M., Kainulainen P., Holopainen J.K. (2004). Significance of wood terpenoids in the resistance of scots pine provenances against the old house borer, *Hylotrupes bajulus*, and brown-rot fungus, *Coniophora puteana*. J. Chem. Ecol..

[41] Higgs M.D., Evans D.A. (1978). Chemical mediators in the oviposition behaviour of the house longhorn beetle, *Hylotrupes bajulus*. Experientia.

